# Exploiting lipotoxicity for the treatment of liver cancer

**DOI:** 10.1038/s41416-021-01479-7

**Published:** 2021-07-23

**Authors:** Ramona Rudalska, Lars Zender, Daniel Dauch

**Affiliations:** 1 Department of Medical Oncology and Pneumology, University Hospital Tuebingen, Tuebingen, Germany; 2 German Cancer Research Consortium (DKTK), German Cancer Research Center (DKFZ), Heidelberg, Germany; 3 iFIT Cluster of Excellence EXC 2180 “Image-Guided and Functionally Instructed Tumor Therapies”, University of Tuebingen, Tuebingen, Germany

**Keywords:** Targeted therapies, Hepatocellular carcinoma

## Abstract

Metabolic alterations occur frequently in solid tumours, but metabolic cancer therapies are limited by the complexity and plasticity of metabolic networks. We could recently show that activation of the liver X receptor alpha (LXRα) and inhibition of a Raf-1-SCD1 protein complex induces an intracellular accumulation of saturated free fatty acids leading to lethal lipotoxicity in tumour cells and allows for an efficient treatment of liver carcinomas.

Solid tumours frequently accumulate alterations in metabolic pathways during development [1, 2]. A metabolic reprogramming may fuel tumour growth due to the specific biosynthetic demands of cancer cells e.g. an increased requirement for lipids for membrane biosynthesis [3]. Furthermore, cancer cells may need to adapt their metabolism to a specific tumour microenvironment and hypoxia [1, 2]. Since metabolic reprogramming in tumour cells may induce dependencies on specific metabolic cascades, therapeutic concepts that exploit metabolic vulnerabilities of tumors have gained major interest in cancer research [1, 2]. Especially, enzymes involved in the de novo synthesis of lipids and their precursors such as fatty acid synthase (FASN), stearoyl-CoA desaturase 1 (SCD1), ATP-citrate lyase (ACLY) and acyl-CoA synthetase short chain family member 2 (ACSS2) represent potential targets for therapeutic approaches [3]. A pharmacological FASN inhibitor (TVB-2640) is currently being tested in a phase II clinical trial against *KRAS* mutated Non-Small Cell Lung Carcinomas (NCT03808558).

However, the development of cancer therapies that are based on the inhibition of important biosynthetic pathways is often impaired by the diversity and plasticity of metabolic networks [1–3]. As a consequence, only a subfraction of tumours may respond to such therapies and a patient selection might be required prior to treatment onset. Furthermore, acquired therapy resistance can rapidly evolve and limit metabolic therapies by activation of compensatory pathways or by an intensified exchange of metabolites between cancer cells and the tumour environment [1, 3].

Notably, metabolic adaptations may render tumour cells also sensitive to toxic metabolites such as reactive oxygen species, trans- or saturated fatty acids [4–6]. A therapy that exploits an intracellular accumulation of toxic metabolites may be specific to tumour cells and independent of the individual tumour metabolism. Therefore, more patients may benefit from such a therapeutic approach.

We could recently show that activation of de novo fatty acid synthesis in combination with an inhibition of fatty acid desaturation results in an accumulation of saturated fatty acids in hepatocellular carcinoma (HCC) cells. Such an accumulation of toxic saturated fatty acids triggers severe lipotoxicity and apoptosis in cancer cells and allows for an efficient treatment of liver tumours with different genetic and metabolic makeups [7].

In order to induce de novo fatty acid synthesis, we exposed HCC cells to synthetic activators of the liver X receptor alpha (LXRα). LXRα forms a heterodimer with retinoid X receptor (RXR) on the LXR response element and controls the expression of genes involved in fatty acid and cholesterol metabolism [8, 9]. The LXR-RXR heterodimer exists either in a basal state where its transcriptional activity is restricted by co-repressors, or in an active state, which is triggered by natural (oxysterols) or synthetic (T0901317, GW3965) ligands and results in the binding of co-activators [8, 9]. Upon activation, LXRα induces expression of sterol regulatory element binding transcription factor 1 (*SREBF1*), acetyl-CoA carboxylase alpha (*ACACA*) and *FASN*, which in turn produces C16:0 and C18:0 fatty acids in presence of their metabolic substrates [10].

These substrates, such as Acetyl-CoA, ATP and NADPH, are often abundant in tumour cells [3]. However, a pharmacological activation of LXRα alone does not lead to lipotoxicity and cell death in cancer cells, since saturated C16/C18 fatty acids are rapidly desaturated by SCD1 (another downstream target of LXRα [10]) to nontoxic mono-unsaturated fatty acids (Fig. 1a). Surprisingly, we identified the RAF proto-oncogene serine/threonine-protein kinase (Raf-1) as a critical activator of SCD1 in HCC cells. We found that Raf-1, an important member of the Ras/MAPK pathway, directly interacts with the desaturase and delays the turnover of SCD1 [7], a short-lived protein with a half-life of 2 to 4 hours [11]. We assume that this protein-protein interaction increases the capability of cancer cells to desaturate toxic saturated fatty acids, highlighting Raf-1 as an important regulator of lipid metabolism in cancer (Fig. 1a).

**Fig. 1:**
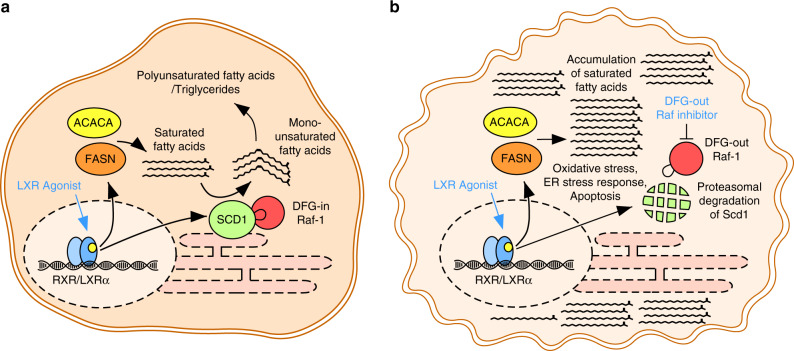
Induction of lipotoxicity for the treatment of liver cancer. **a** Activation of LXRα alone induces de novo fatty acid synthesis in cancer cells. However, saturated fatty acids are rapidly desaturated by SCD1 due to a Raf-1 mediated stabilization of the enzyme. **b** Inhibition of Raf-1 by DFG-out Raf inhibitors induces proteasomal degradation of SCD1 leading to an intracellular accumulation of toxic saturated fatty acids, lipotoxicity and consequently to apoptosis of liver cancer cells upon LXRα activation.

In order to target the Raf-1-SCD1 protein complex in HCC, we treated liver cancer cells with different Raf inhibitors. While compounds that induce an “in” conformation of the DFG motif in the kinase (e.g. dabrafenib, SB590885) [12] were not able to affect the Raf-1-SCD1 interaction, DFG-out Raf inhibitors such as sorafenib or BI-882370 [12] efficiently disrupt this protein complex leading to rapid proteasomal degradation of SCD1 (Fig. 1b). In line with these data, a combinatorial therapy comprising an LXRα agonist and a DFG-out Raf inhibitor triggers a strong intracellular accumulation of saturated fatty acids in liver cancer cells. Such an accumulation of toxic lipids results in oxidative stress, a critical ER stress response (activation of PERK and its downstream targets eIF2α, GADD34 and CHOP), a formation of lipid droplets and subsequently in apoptosis of liver cancer cells [7] (Fig. 1b). Treatment studies in autochthonous, transposon-based liver cancer mouse models [13] and xenograft models of human HCC revealed, that this combinatorial therapy is highly effective in order to suppress genetically and metabolically different liver carcinomas and to significantly extend the survival of tumour bearing animals.

HCC represents a leading cause of cancer-related death worldwide and it is expected that the HCC incidence will further rise in the future due to increasing rates of fatty liver diseases such as non-alcoholic steatohepatitis (NASH) [14]. HCCs exhibit strong resistance to cytotoxic and targeted therapies [14, 15] and only a subtype of tumours responds to recently developed immunotherapies [16]. Especially, the increasing numbers of NASH-associated HCCs are largely resistant to checkpoint inhibitors [16, 17]. Importantly, our novel lipotoxic therapy was effective against NASH-driven HCCs, indicating a strong potential for the treatment of NASH-HCC patients in the future. Since this therapy was well tolerated by mice, even by animals that suffer from severe NASH [7], we believe that a therapeutic window for the treatment of human liver cancer patients exits.

The DFG-out Raf inhibitor sorafenib is routinely used for the treatment of liver cancer patients [15, 18] and could be therefore easily applied as a component of our combinatorial lipotoxic therapy. Since LXRα agonists are not yet approved for the treatment of patients, the development of potent and specific LXRα agonists might be an important task for the future in order to translate our findings into the clinic.

## Data Availability

Not applicable.
